# EGFR-TKIs联合伽玛刀治疗*EGFR*突变的肺腺癌伴脑转移的预后分析

**DOI:** 10.3779/j.issn.1009-3419.2019.05.08

**Published:** 2019-05-20

**Authors:** 丽敏 陈, 梦姣 傅, 建娅 周, 一楠 姚, 建英 周

**Affiliations:** 310003 杭州，浙江大学医学院附属第一医院呼吸内科 Respiratory Department, the First Affiliated Hospital, College of Medicine, Zhejiang University, Hangzhou 310003, China

**Keywords:** 表皮生长因子受体酪氨酸激酶抑制剂, 伽玛刀, 肺腺癌, 脑转移, 预后因素, Epidermal growth factor receptor-tyrosine kinase inhibitor (EGFR-TKI), Gamma knife radiosurgery, Lung adenocarcinoma, Brain metastasis, Prognostic factors

## Abstract

**背景与目的:**

晚期表皮生长因子受体（epidermal growth factor receptor, EGFR）基因突变的肺腺癌在初诊或治疗过程中脑转移的总发生率高，局部治疗联合系统性靶向治疗可能是更佳策略。本研究拟探讨分析EGFR酪氨酸激酶抑制剂（tyrosine kinase inhibitors, TKIs）联合头颅伽玛刀治疗*EGFR*突变的肺腺癌伴脑转移患者的疗效及预后因素。

**方法:**

回顾性收集*EGFR*基因突变的肺腺癌、在初诊即存在脑转移或在EGFR-TKIs治疗过程中肺部病灶稳定而出现脑转移、接受一线口服EGFR-TKIs靶向治疗联合脑部伽玛刀局部治疗的患者，评价EGFR-TKIs联合头颅伽马刀治疗对颅内病灶的疗效，随访并分析颅内无进展生存时间（intracranial progression free survival, i-PFS），探索*EGFR*突变肺腺癌伴脑转移的预后因素。

**结果:**

共纳入74例患者，其中位i-PFS为14.7个月，1年无颅内进展率为58.5%，2年无颅内进展率为22.2%。颅内病灶与肺部病灶具有相近的进展时间。单因素生存分析显示，初诊癌胚抗原（carcinoembryonic antigen, CEA）水平 < 10 ng/mL（16.9个月*vs* 12.6个月，*P*=0.012）、颅内病灶长径 < 2 cm（15.4个月*vs* 10.8个月，*P*=0.021）、肺癌脑转移分级预后系统（lung graded prognostic assessment, Lung-molGPA）评分 > 3（15个月*vs* 12.6个月，*P*=0.041）的患者具有更长的i-PFS。多因素分析显示初诊时CEA≥10 ng/mL和颅内病灶≥2 cm是i-PFS的不良预后因素。

**结论:**

EGFR-TKIs联合伽玛刀局部治疗对*EGFR*突变肺腺癌伴脑转移患者的颅内病灶具有良好的疗效。初诊时CEA水平≥10 ng/mL、颅内病灶≥2 cm是接受EGFR-TKIs联合伽马刀治疗的脑转移肺腺癌患者的不良预后因素。

## Abstract (en)

**Background and objective:**

Advanced epidermal growth factor receptor (*EGFR*)-mutant lung adenocarcinoma had a high overall incidence of brain metastasis during the full course, and local brain radiotherapy combined with systemic targeted therapy may be a better strategy. This study aimed to identify the prognostic factors of *EGFR*-mutant brain-metastatic lung adenocarcinoma patients who received EGFR-tyrosine kinase inhibitors (EGFR-TKIs) in combination with gamma knife radiosurgery.

**Methods:**

Retrospective analysis of *EGFR*-mutant lung adenocarcinoma patients with brain metastases which developed at initial diagnosis or during EGFR-TKIs treatment period were performed. Intracranial progression free survival (PFS) was statistically analyzed between different subgroups to find out the prognostic factors including gender, age, smoking history, extracranial metastasis, *EGFR* mutation type, size and number of intracranial lesions, carcino-embryonic antigen (CEA) level, lung-molGPA score and so on.

**Results:**

A total of 74 *EGFR*-mutant brain-metastatic lung adenocarcinoma patients were enrolled in this study, with median intracranial PFS of 14.7 months. One-year intracranial-progression-free rate was 58.5%, and two-year rate was 22.2%. Univariate survival analysis showed that patients with lower CEA level at initial diagnosis (< 10 ng/L)(16.9 months *vs* 12.6 months, *P*=0.012) and smaller intracranial lesions (< 2 cm)(15.4 months *vs* 10.8 months, *P*=0.021) and higher lung-molGPA score (> 3)(15 months *vs* 12.6 months, *P*=0.041) were prone to have a superior intracranial PFS. Multivariate analysis showed that CEA≥10 ng/mL and intracranial lesion≥2 cm were the independent risk factors of intracranial PFS.

**Conclusion:**

EGFR-TKIs in combination with gamma knife radiosurgery was an efficient treatment option to control the cranial tumor lesion. CEA≥10 μg/L at initial diagnosis and intracranial lesion≥2 cm were the risk factors of *EGFR*-mutant brain-metastatic lung adenocarcinoma patients receiving EGFR-TKIs in combination with gamma knife radiosurgery.

肺癌是全球发病率最高的恶性肿瘤，非小细胞肺癌（non-small cell lung cancer, NSCLC）约占总体肺癌的80%，其中腺癌约占40%。晚期肺腺癌在初诊或治疗过程中脑转移的总发生率在50%以上^[1]^，其预后差，自然中位生存期仅4周-6周^[2]^，即使经过全脑放疗，其中位总生存期（overall survival, OS）也仅为3个月-6个月，1年生存率仅10%-15%^[3]^。近10余年来由于肺癌靶向治疗的进展，驱动基因阳性的NSCLC晚期患者的生存已得到显著改善。表皮生长因子受体酪氨酸激酶抑制剂（epidermal growth factor receptor-tyrosine kinase inhibitors, EGFR-TKIs）对于*EGFR*基因突变的NSCLC的有效率达70%以上，中位OS延长至12.9个月-21.9个月^[4-7]^。对于EGFR突变的肺腺癌伴脑转移患者，EGFR-TKIs也展示出了一定的疗效。然而由于现有的一代EGFR-TKIs存在的颅内血药浓度不足的缺陷，即使在靶向治疗过程中，患者仍会出现颅内病灶的进展，局部治疗联合系统性靶向治疗可能是更佳策略。脑转移的局部治疗包括手术、全脑放疗（whole brain radiation therapy, WBRT）以及立体定向放射（stereotactic radiosurgery，SRS，伽马刀是其中一种）。目前已有多项研究显示EGFR-TKIs联合WBRT可延长脑转移患者的OS。对于EGFR-TKIs联合SRS的疗效及预后分析的研究则较少^[8]^，相关数据缺乏，因此有必要进一步分析EGFR-TKIs联合伽马刀对肺腺癌脑转移患者的颅内病灶的控制情况并探索其相关的预后因素，为临床诊疗提供重要的参考借鉴。

## 1 资料和方法

### 1.1 一般资料

选取2012年2月-2017年1月在浙江大学医学院附属第一医院收治的、经病理确诊、*EGFR*突变的肺腺癌伴脑转移患者，包括初诊即存在脑转移或治EGFR-TKIs治疗过程中肺部病灶稳定而出现颅内转移的患者。入选标准：①以EGFR-TKIs作为一线治疗；②在发现脑转移病灶起3个月内进行脑部伽马刀治疗；③头颅磁共振成像（magnetic resonance imaging, MRI）示脑部单个转移灶≤5 cm，或脑部转移灶数量≤6个且最大移灶的直径≤3 cm；④美国东部肿瘤协作组评分（Eastern Cooperative Oncology Group performance status, ECOG PS）≤2分；⑤定期规律随访，肺部CT及头颅MRI检查间隔时间≤3个月。排除标准：①*EGFR* T790M原发突变；②合并严重全身性疾病；③除伽马刀外曾接受其他脑部局部治疗。收集患者的性别、年龄、吸烟史、ECOG评分、基因状态、颅外转移、脑转移发生时刻、肺部病灶大小、颅内病灶大小与数量、神经症状、初诊时癌胚抗原（carcino-embryonic antigen, CEA）水平、Lung-molGPA评分等资料。Lung-molGPA根据患者的年龄、卡氏体力状态评分（karnofsky performance status, KPS）、是否有颅外转移、脑转移数量、EGFR及间变性淋巴瘤激酶（anaplastic lymphoma kinase, *ALK*）基因状态等因素评分^[9]^。

### 1.2 治疗方法

#### 1.2.1 立体定向放疗

采用增强MRI扫描进行定位，使用Leksell-Perfxion型头部伽玛刀进行治疗，遵循RTOG90-05方案控制靶区周围剂量，用50%等剂量曲线进行包绕，期间如出现头痛呕吐等脑水肿表现，予甘露醇、糖皮质激素脱水治疗。

#### 1.2.2 EGFR-TKIs靶向治疗

所有患者接受吉非替尼250 mg每日一次或埃克替尼125 mg每日三次或厄洛替尼150 mg每日一次口服，治疗过程中可根据患者出现的不良事件作相应的剂量微调。

### 1.3 疗效评价

伽玛刀治疗结束后4周-6周复查头颅MRI及胸部CT，按照实体瘤疗效评价标准（Response Evaluation Criteria in Solid Tumors, RECIST）1.1疗效评价标准评估疗效，分为完全缓解（complete response, CR）、部分缓解（partial response, PR）、稳定（stable disease, SD）、疾病进展（progressive disease, PD）。随访并计算颅内客观缓解率（objective response rate, ORR）、颅内无进展生存时间（intracranial progression free survival, i-PFS）和肺部无进展生存时间（lung progression free survival, l-PFS）。I-PFS定义为接受TKIs治疗第1天至颅内肿瘤进展或随访截止的时间，l-PFS定义为接受TKIs治疗第1天至肺部病灶进展或随访截止的时间。随访截至2017年11月10日，随访方式包括病历回顾、门诊随访及电话随访。不良事件根据美国国立癌症研究院通用毒性标准（National Cancer Institute Common Terminology Criteria for Adverse Events, NCI-CTCAE 4.03）进行评价。

### 1.4 统计学方法

应用SPSS 20.0分析数据，生存分析采用*Kaplan*-*Meier*法，组间差异采用*Log*-*rank*检验。多因素分析采用*Cox*比例风险模型。相关性分析采用*Pearson*相关。以*P* < 0.05为差异有统计学意义。

## 2 结果

### 2.1 患者基本资料

根据入排标准，共纳入74例患者，其中男性30例，女性44例；年龄36岁-79岁，中位年龄59岁。全组患者的临床特征资料见表 1。74例患者脑转移灶数量1个-6个不等，共检出转移灶188个。伽玛刀治疗周边照射剂量为12 Gy-22 Gy，分单次（小病灶）或分多次（大病灶或位于功能区病灶）照射，中位剂量18 Gy。21例初治无脑转移患者，其中11例在中位服药13.1个月后出现孤立脑转移。53例初诊即存在脑转移的患者，其中16例在中位服药12.4个月后出现新的脑转移病灶。

**1:** 纳入的74例肺腺癌伴脑转移患者的临床特征 Clinical characteristics of the enrolled 74 cases

Clinical features	*n*	Percentage
Gender		
Male	30	40.5%
Female	44	59.5%
Age		
< 60	40	54.1%
≥60	34	45.9%
Smoking history		
Yes	20	27.0%
No	54	73.0%
ECOG score		
0-1	55	74.3%
2	19	25.7%
Extracranial metastasis		
Yes	23	31.1%
No	51	68.9%
Lymph-node stage		
N3	19	25.7%
N0/N1/N2	55	74.3%
Size of lung lesion		
< 3 cm	36	48.6%
≥3 cm	38	51.4%
*EGFR* mutation type		
21 L858R mutation	36	48.6%
19 Exon deletion	38	51.4%
Size of intracranial lesion		
< 2 cm	55	74.3%
≥2 cm	19	25.7%
Number of intracranial lesions		
Single	30	40.5%
Multiple	44	59.5%
CEA level at initial diagnosis		
< 10 ng/mL	34	45.9%
≥10 ng/mL	40	54.1%
Neurologic symptoms		
Yes	24	32.4%
No	50	67.6%
Synchronous brain metastasis		
Yes	53	71.6%
No	21	28.4%
Site of first progression		
Lung	33	44.6%
Brain	41	55.4%
Lung-molGPA score		
0-1	0	0
1.5-2	7	9.5%
2.5-3	34	45.9%
3.5-4	33	44.6%
EGFR: epidermal growth factor receptor; ECOG: Eastern Cooperative Oncology Group; CEA: carcino-embryonic antigen.		

### 2.2 病灶缓解情况

在全体患者中，发生失访4例，意外死亡1例。治疗结束后4周-6周首次评价颅内病灶的缓解情况，CR 6例，PR 48例，SD 20例，PD 0例。颅内病灶ORR为73.0%。

### 2.3 颅内中位PFS

至随访结束，共有56例患者发生颅内病灶进展，尚有18例患者未发生颅内病灶进展，中位i-PFS为14.7个月（95%CI: 12.1个月-17.2个月；图 1A）。1年颅内无进展生存率58.5%，2年颅内无进展生存率为22.2%。

**1:**
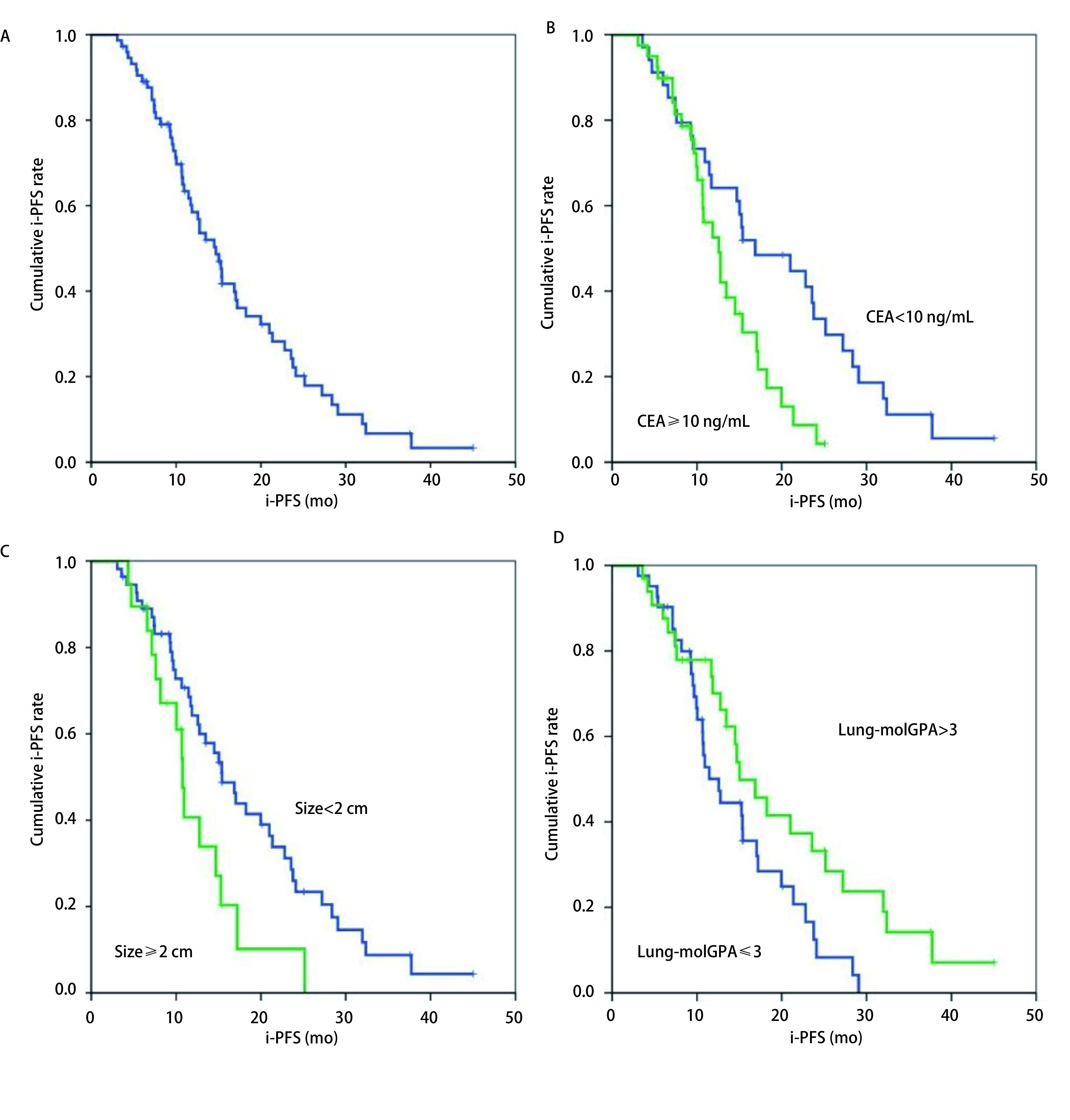
生存曲线 Survival curves

对i-PFS进行单因素分析显示，初诊CEA水平 < 10 ng/L的患者的i-PFS优于初诊CEA水平≥10 ng/L患者（16.9个月*vs* 12.6个月，*P*=0.012；表 2，图 1B），颅内病灶长径 < 2 cm的患者的i-PFS优于颅内病灶长径≥2 cm（15.4个月*vs* 10.8个月，*P*=0.021；表 2，图 1C），Lung-molGPA评分 > 3分的患者优于≤3分的患者（15个月*vs* 12.6个月，*P*=0.041；表 2，图 1D）。

**2:** 患者临床特征与i-PFS相关性的单因素分析 Univariate analysis of correlation between clinical characteristics and i-PFS

Clinical features	*n*	Median i-PFS (mo)	95%CI	*P*
Gender				0.599
Male	30	12.8	11.3-14.2	
Female	44	15.4	12.3-18.5	
Smoking history				0.817
Yes	20	12.8	11.6-14.0	
No	54	15.3	11.9-18.7	
Age (yr)				0.983
< 60	40	15.0	10.2-19.9	
≥60	34	14.5	11.3-17.7	
Extracranial metastasis				0.097
Yes	23	11.5	8.7-14.2	
No	51	15.0	11.2-18.9	
Lymph-node stage				0.694
N3	19	15.3	12.1-18.5	
N0/N1/N2	55	14.5	10.8-18.2	
Size of lung lesion				0.195
< 3 cm	36	15.4	12.4-18.4	
≥3 cm	38	12.6	10.5-14.7	
*EGFR* mutation type				0.841
21 L858R mutation	36	13.5	8.7-18.2	
19 Exon deletion	38	15.3	11.3-19.3	
Size of intracranial lesion				0.021*
< 2 cm	55	15.4	12.3-18.5	
≥2 cm	19	10.8	9.7-11.8	
Number of intracranial lesions				0.660
Single	30	16.9	7.2-26.5	
Multiple	44	13.5	9.9-17.0	
CEA level at initial diagnosis				0.012*
< 10 ng/mL	34	16.9	9.2-24.6	
≥10 ng/mL	40	12.6	10.0-15.0	
Neurologic symptoms				0.265
Yes	24	16.9	12.4-21.3	
No	50	13.5	9.6-17.4	
Site of first progression				0.309
Lung	33	15.0	11.9-18.2	
Brain	41	14.5	9.0-20.0	
Synchronous brain metastasis				0.596
Yes	53	14.5	12.0-17.0	
No	21	20.0	6.0-34.0	
Lung-molGPA score				0.041*
≤3	41	12.6	9.7-15.5	
> 3	33	15.0	10.7-19.3	
^*^P < 0.05. i-PFS: intracranial progression free survival.				

将单因素分析中*P* < 0.2的因素进行*Cox*多因素分析显示初诊时CEA水平和颅内病灶大小是i-PFS的不良预后因素。初诊CEA水平 < 10 ng/L（OR=1.999, 95%CI: 1.094-3.653, *P*=0.024）、颅内病灶长径 < 2 cm（OR=2.088, 95%CI: 1.100-3.963, *P*=0.024）的患者能够获得更长的i-PFS（表 3）。

**3:** 患者临床特征与i-PFS相关性的多因素分析 Multivariate analysis of correlation between clinical characteristics and i-PFS

Factor	OR	95%CI	*P*
Extracranial metastasis	1.057	0.485-2.304	0.888
Size of lung lesion	1.333	0.751-2.364	0.326
Size of intracranial lesion	2.088	1.100-3.963	0.024^*^
Number of intracranial lesions	0.985	0.492-1.973	0.967
CEA level at initial diagnosis	1.999	1.094-3.653	0.024^*^
Lung-molGPA score	0.758	0.362-1.586	0.462
^*^P < 0.05			

### 2.4 颅内PFS和肺部PFS的相关性

中位i-PFS为14.7个月，中位l-PFS为19.4个月。将i-PFS和l-PFS进行*Pearson*相关性分析，发现两者之间存在正相关，相关性系数为0.685（*P* < 0.001）。肺内病灶与颅内病灶进展的时间差（影像学上分别显示肺内病灶进展及颅内病灶进展的日期之差）的中位数为10 d，四分位数间距为-23.3 d-125.3 d，说明颅内进展与肺部病灶几乎同时进展。

## 3 讨论

目前有多项研究认为*EGFR*突变是肺癌患者发生脑转移的危险因素。Hsu等^[10]^针对543例NSCLC患者进行的一项回顾性研究显示，*EGFR*突变患者较野生型患者有更高的脑转移发生率（39.2% *vs* 28.2%, *P*=0.038）。Luo等^[11]^分析了374例肺腺癌患者，提示EGFR 19外显子突变患者的脑转移发生率为48.1%，明显高于野生型患者。Han等^[12]^的研究也证实了*EGFR*突变是脑转移的独立危险因素。因此，对于*EGFR*突变的肺腺癌患者，脑转移病灶的管理至关重要。然而，也有不少研究证实，接受了分子靶向治疗联合局部治疗的患者较EGFR野生型患者有更好的预后。Yang等^[13]^研究认为在EGFR-TKIs联合脑部SRS患者中，*EGFR*突变患者较野生型患者有更高的缓解率、更长的脑部复发时间及i-PFS。最近的一项研究^[14]^显示早期行SRS对于*EGFR*突变型伴局限脑转移患者是微创且有效的方法，而亚组分析显示EGFR-TKIs治疗作为一线治疗的人群有更长的生存时间和更持久的颅内控制时间。

伽玛刀属于SRS的一种，与全脑普通放疗相比，伽玛刀能够精确照射肿瘤靶区，在瘤体内射线剂量大，可使肿瘤体积迅速缩小，甚至消失，而瘤周正常组织受照射量小，因此可以提高局部控制率，并减轻包括脑水肿、脑神经功能损害、认知功能障碍等在内的不良反应。目前单纯头部伽玛刀或联合全脑放疗已广泛应用于颅内转移瘤的治疗。

本研究针对74例*EGFR*突变肺腺癌伴脑转移患者，采用伽玛刀治疗脑部病灶，同时联合TKIs全身治疗，对患者进行预后的分析，以进一步探究伽玛刀联合EGFR-TKIs治疗*EGFR*突变的肺腺癌脑转移的疗效、安全性及预后因素。本研究纳入的74例患者的中位颅内无进展生存时间为14.7个月。初诊时同时伴随脑转移的患者中位i-PFS为14.5个月，而治疗过程中出现脑转移的患者中位i-PFS为20个月，两者无统计学差异（*P*=0.596），说明EGFR-TKIs系统性治疗联合伽马刀局部治疗在不同时刻出现脑转移的患者中具有相近的疗效。同类研究表明，在初诊即伴脑转移的患者中，先SRS后TKI组的中位i-PFS为23个月，而先EGFR-TKIs再SRS（即延迟放疗）组的中位i-PFS为17个月^[15]^，均高于本研究的i-PFS结果，其可能原因是本研究在纳入患者时并未排除随访时间 < 6个月的患者，且总随访时间较该研究偏短，部分患者未达观察终点。对于治疗过程中出现的颅内病灶的进展，有两种可能的机制：一方面，肿瘤细胞发生继发性耐药突变，对TKIs产生耐药，肿瘤未得到有效控制而产生新的转移灶或是原有颅内病灶增大；另一方面，肺内原发肿瘤得到有效控制，而颅内因血药浓度太低无法使原有病灶缩小，也不能有效阻止血液中的肿瘤细胞在脑内定居、增殖进而形成新的转移灶。在前者情况下，更换抑制耐药突变的新一代EGFR-TKIs是首要选择，而在后者情况下，继续予当前的TKIs治疗同时联合颅内病灶局部治疗（主要为放疗）才是使患者获益最大的方案。

本研究的相关性研究部分显示，i-PFS和l-PFS正相关，肺脑进展时间差的中位数为10 d，四分位数间距为-23.3 d-125.3 d，考虑到患者在随访过程中肺部及头颅影像检查的时间差，提示多数患者颅内病灶与肺部病灶几乎同时进展。而肺部病灶的进展多由于肿瘤细胞产生继发性耐药突变，颅内进展随之发生，说明在耐药时刻前，TKIs联合伽马刀对颅内病灶尚有较强的控制力度。进展之后，更换抑制耐药突变的、颅内血药浓度更高的新一代TKIs，同时再次进行伽马刀治疗，可能能使患者取得更多的生存获益。

颅内病灶 < 2 cm（*P*=0.021）、初诊时CEA < 10 ng/mL（*P*=0.012）、Lung-molGPA > 3（*P*=0.041）的患者较颅内病灶≥2 cm、初诊CEA≥10 ng/mL、Lung-molGPA≤3的患者有更长的颅内PFS。而年龄、性别、吸烟史、肺部病灶大小、*EGFR*突变类型、颅内病灶数量、是否有神经症状等因素对患者的中位颅内无进展生存时间无显著影响。进一步的多因素分析显示，颅内病灶大小、初诊时CEA水平也是i-PFS的不良预后因素。以上两个因素均反映了机体的肿瘤负荷量，说明肿瘤负荷显著影响颅内病灶预后。有高肿瘤负荷者，即使接受TKIs治疗，仍有更高的概率发生新的血道转移。此外，由于TKIs颅内血药浓度不足，不足以抑制肿瘤细胞在颅内的定居、增殖进而形成新的转移灶，故颅内高瘤负荷患者更易出现颅内进展。对于这部分患者，应缩短MRI复查时间，密切关注病灶变化并给予及时的处理。

不良反应方面，本研究纳入的患者主要有头痛头昏、恶心呕吐、肢体活动障碍、均经脱水降颅压等对症治疗后顺利完成治疗，无一例发生III度以上不良反应，说明伽马刀具有较高的安全性。因此，EGFR-TKIs联合伽玛刀治疗*EGFR*突变肺腺癌脑转移安全有效，可作为*EGFR*突变的肺腺癌伴脑转移患者的临床选择。

本研究中纳入的患者在接受伽马刀的治疗时机上存在一定的差异。对于初诊即伴脑转移的患者，多数是在发现脑转移之初半月内即接受伽马刀治疗，但也有少部分患者在发现颅内病灶并接受TKIs治疗2个月-3个月后才进行颅内病灶的伽马刀治疗。因样本量较少，无法完成有效统计，但初步统计发现，两组在i-PFS上不存在显著差异。既往也有研究对SRS治疗的时机选择进行了探索。Magnuson^[16]^比较了针对*EGFR*突变的NSCLC脑转移患者先行TKIs治疗后行放疗（即延迟放疗）和TKIs联合同步放疗的预后差异，结果显示TKIs联合同步放疗组有更长的OS及颅内PFS。Wang等^[8]^研究则发现EGFR-TKIs同步或序贯SRS获得了相近的疗效。SRS治疗的最佳时机目前尚无定论，尚需进一步前瞻性、大样本、长随访时间的研究以明确。

本研究发现，肿瘤负荷（颅内病灶大小、CEA）是影响*EGFR*突变肺腺癌伴脑转移患者颅内控制情况的预后因素，对于高瘤荷患者建议缩短头颅MRI复查时间。本研究为回顾性研究，样本量略偏少，且存在一定的偏倚，因此有待于设计严谨的大型前瞻性临床研究来进一步证实上述研究结论。此外，伽马刀治疗的最佳时机也待进一步研究来验证。
